# Challenges and opportunities in mobile e-coaching

**DOI:** 10.3389/fdgth.2023.1304089

**Published:** 2024-01-30

**Authors:** Jan-Willem J. R. van 't Klooster, Lucia M. Rabago Mayer, Bart Klaassen, Saskia M. Kelders

**Affiliations:** Faculty of Behavioural, Management and Social Sciences, University of Twente, Enschede, Netherlands

**Keywords:** e-health, e-coaching, remote monitoring, JITAI, experience sample methodology

## Abstract

**Background:**

Mobile e-health technologies have proven to provide tailored assessment, intervention, and coaching capabilities for various usage scenarios. Thanks to their spread and adoption, smartphones are one of the most important carriers for such applications.

**Problem:**

However, the process of design, realization, evaluation, and implementation of these e-health solutions is wicked and challenging, requiring multiple stakeholders and expertise.

**Method:**

Here, we present a tailorable intervention and interaction e-health solution that allows rapid prototyping, development, and evaluation of e-health interventions at scale. This platform allows researchers and clinicians to develop ecological momentary assessment, just-in-time adaptive interventions, ecological momentary intervention, cohort studies, and e-coaching and personalized interventions quickly, with no-code, and in a scalable way.

**Result:**

The Twente Intervention and Interaction Instrument (TIIM) has been used by over 320 researchers in the last decade. We present the ecosystem and synthesize the main scientific output from clinical and research studies in different fields.

**Discussion:**

The importance of mobile e-coaching for prediction, management, and prevention of adverse health outcomes is increasing. A profound e-health development strategyand strategic, technical, and operational investments are needed to prototype, develop, implement, and evaluate e-health solutions. TIIM ecosystem has proven to support these processes. This paper ends with the main research opportunities in mobile coaching, including intervention mechanisms, fine-grained monitoring, and inclusion of objective biomarker data.

## Introduction

In the current and next decade, multiple healthcare challenges are pressing upon us because of an aging population. The prevalence of morbidities, including cancer, stroke, and cardiovascular and neurodegenerative diseases, is increasing; the same holds for injuries due to aging, overweight, and diabetes. At the same time, qualified personnel is scarce and trained workers in the healthcare sector itself are lacking.

Hence, it is necessary to develop and implement cost and resource-saving digital solutions. Mobile e-health technologies have proven to provide capabilities for various usage scenarios (1). This includes remote monitoring, physiological assessment, behavioral intervention, and coaching capabilities. In the last few decades, e-health solutions have become multimedial, interactive, configurable, tailorable, personalizable, and—in some cases—even smart. A wide range of (clinical) cases allowed for the assessment, intervention, and coaching of patients (2, 3).

Thanks to their spread and adoption, smartphones are an important driver for such applications. They function as a personal gateway for data transport, user interface, and prompting devices. However, the process of design, realization, evaluation, and implementation of these e-health solutions is wicked, challenging, and cost-intensive, and it requires multiple stakeholders and expertise (1, 4).

In this contribution, we specifically focus on ecological momentary assessment (EMA) and ecological momentary intervention (EMI) systems (5, 6). These systems aim at providing a detailed picture of the patient/participant through frequent (random) smartphone prompting with short questionnaires (EMA); eventually combined with intervention or advice (EMI). A recent development in the latter is Just-In-Time Adaptive Intervention (JITAI) (7–9), in which the intervention or advice is calculated, communicated, and personalized online, i.e., not beforehand, enabling real-time electronic coaching through a digital coach.

### Generations ES

In the last few decades, various tailored EMA/EMI systems were developed and tested, including non-specific, flexible, and configurable ones (5). Although not a strict classification, in general, they can be divided into four somewhat overlapping generations:

*First generation*: a linear path along which the assessment of questions is set up, which is defined *a priori* by the researcher. Often, the applications are bound to specific usage scenarios, with limited room for deviation from the intended usage pathway.
*Second generation*: schedulable assessments are possible that are tailorable to end users, contain multimedia questions, and hence greatly improve the flexibility of the instrument for various usage scenarios.
*Third generation:* such systems contain interactive functions that greatly improve the intervention aspect of ambulatory assessment. Live calculation with variables, interactive feedback, and multimodal input/output is available.
*Fourth generation:* The latest generation of systems includes biomarkers such as pulse rate, accelerometry (10), amount of steps, and sleep data and makes use of this to get (a) fine-grained detailed qualitative *and* quantitative measurement results from participants or patients; (b) and/or give adaptive Just-In-Time feedback based on configurable settings.

Currently, limited tooling exists that offers configurable third/fourth-generation functionality. In many cases, systems are tailored to specific diseases or interventions, which may be related to scope and project funding reasons. However, there are also non-specific systems, including Data Ethica, Lime Survey, and MPath. Managed by commercial vendors, these systems are under continuous development and improvement. As such, the functionality, improvements, and security are dictated by vendors and not by the research community. Moreover, vendor lock-in is a risk.

To overcome this risk, address research demands in a way that allows for flexibility, safe (GDPR and ISO7510 conform) data storage and control over the platform, and support for all of the generations mentioned over time in a cost-effective way without the need of programming (no-code solution), we started the development of the Twente Intervention and Interaction Instrument (TIIM) in 2017. The remainder of this article presents TIIM and its main architectural choices, shows a representative selection of research results achieved with it, and sets out further challenges and opportunities.

## TIIM

**Figure 1:**
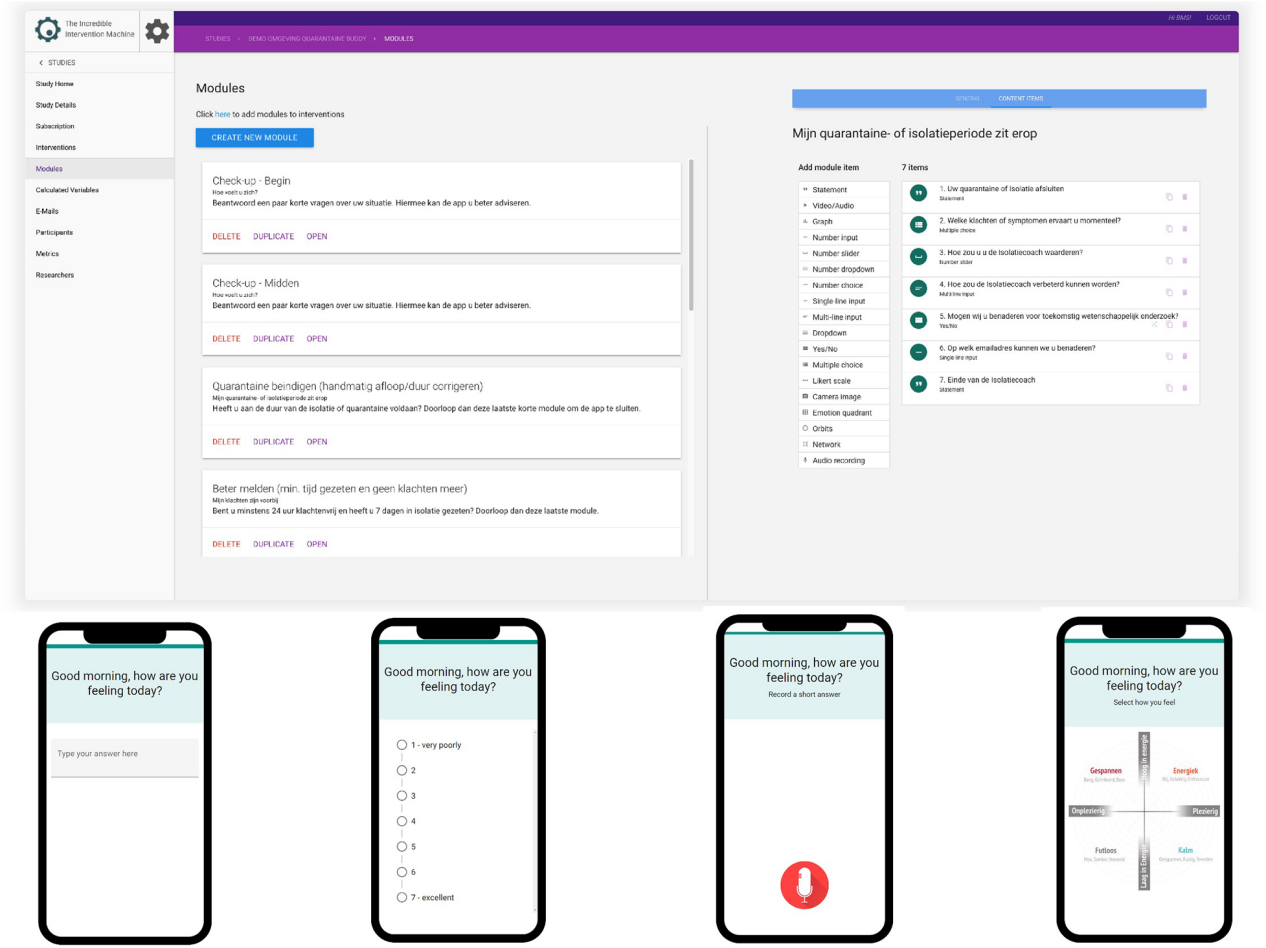
Administration portal (researcher/professional side, top) and front end (participant/patient side, bottom) of TIIM.

TIIM allows researchers and clinicians to define *studies* with one or more different versions of *interventions* (i.e., the concrete package of digital components forming the psychological, behavioral, emotional, or physical intervention to trigger actions). Each intervention consists of *modules* (chapters) with *questions* and/or multimedia informative components. These modules can be scheduled to release in a predefined order or interactively based on answers given by the participant. Where researchers/clinicians operate the backend from a web application, the end users/patients download a free Android/iOS app and join one or more studies using a study code or by scanning a QR code. They can then be (automatically) assigned or included in the interventions or put on a waiting list. The platform allows participants to be reminded by (if need be multiple) push notifications at random or select intervals to fill out scheduled modules once enrolled in a study. Using this approach, the platform allows for mobile questionnaires, A/B testing, multiple cohorts, (app) prototype testing, intervention piloting, EMA/EMI studies, e-learning, and mobile coaching, all without the need to program computer code (no-code solution). Figures 1A,B depict the front end (participant/patient side) and administration portal (researcher/professional side) of TIIM.

It is beyond the scope of this article to discuss all details of TIIM, apart from the available question types also found in other survey software including open/closed questions, multiple choice, drag and drop, slide, number input, text field, dropdown, and image and microphone input. Within the context of this article, there are three more noteworthy aspects helpful in organizing the end user/patient feedback. These are *scheduling and reminding*, *calculating variables*, and collecting *biomarker data*.

### Scheduling and reminding

#### Scheduling of intervention modules

The different parts with information content and questions, i.e., Modules of the Intervention, can be scheduled to be presented to end users (participants). This allows for interactive a dynamic coaching. The options are to schedule:

• On a relative time after the intervention started: This option will enable this module after a certain amount of time has passed since the participant has started the intervention.
• On a relative time after a module has finished: This option will enable this module after a certain amount of time has passed AFTER the participant has completed a different module.
• When a module has not been finished.
• Based on an answer on a module item: This option will enable the module when an item answer condition has been met.
• On a fixed date and time.

Then, the modules can be set to close down:

• Based on an answer on a module item.
• On a fixed date.
• Based on a relative time after a module has started.

This allows for flexibility and configurability in what is being presented to users at which point in time, both at the item and module item to realize tailor-made interventions.

#### Module notifications and reminders

Next to the actual module scheduling, users also can be reminded of modules by (push) notification prompts on their devices using native notifications.

An opening notification can be sent based on an interval or a fixed date. A reminder notification will be sent after the module is available for at least 15 min. A closing notification will be sent 15 min before the module is about to close. A notification may contain custom text. Only one opening and one closing notification can be created. A closing notification can only be created if the module has one of the following closing timing rules: (1) on a fixed date; and (2) based on a relative time after a module has started.

#### Calculated variables

By using calculating variables, equations can be made to dynamically change intervention behavior at the runtime level. For example, it can ask how happy a patient is today on a 1–10 scale for 3 days in three modules. Then, a calculated variable can be configured to display and store a 3-day average. Or, if a patient is feeling well on a given day, the system does not show the remainder of the questions applicable in case they do *not* feel well.

Different functions are available, including the following:

• **“**+**”** gives the addition of variables;
• **“-”** gives the subtraction of variables;
• “*” gives the multiplication of variables;
• **“/”** gives the division of variables;
• **“^”** gives the exponential value of a number;
• **“SUM_ANS(N)”** gives the sum of the different participant responses to the variable;
• **“NUM_PS(N)”** gives the total number of participants for an indicated variable. Example: three participants are assigned to an intervention, this function will return 3;
• **“NUM_P_ANS(N)”** provides the number of participants who answered the indicated variable. For example, If three participants are assigned to an intervention and two give an answer, this function will return 2;
• **“MODE_ANS(N)”** gives the most responded number of the indicated variable. *Note: If there is no mode, this function will return the average*; and
• **“COUNT_ANS(VAR,VAL)”** provides the number of times the value of the responses was given in the indicated variable.

Calculated variables can be used to design timing rules or to give interactive feedback. This functionality allows for an extremely powerful synthesis of user answers and interactivity in designing interventions and feedback.

#### Biomarker data

The TIIM app aims to allow harvesting of health and wellbeing data for research from biomarkers. With an array of powerful features, health data can be monitored, which can expand and enrich qualitative and quantitative research. The following data can be collected by the mobile app.

##### Number of steps in a day

Most mobile phones can track steps on daily steps and provide insightful information about how activity is distributed throughout the day.

##### Pulse rate and Hr(V) indication

Today, users can wear a device providing heart rate measures (pulse rate, pulse rate variability) over the day.

##### Sleep duration and classification

Quality sleep can be monitored by most phones. This means TIIM can access the sleep duration, analyze sleep patterns, and provide a comprehensive view of sleep quality.

The data are obtained by using the integration via Apple Health/Google Fit. This means that any wearable or data can be brought to either mobile app. In this case, participants need to give access to the TIIM and Apple Health and/or Google Fit consent to share the data. This means that the researcher can effortlessly view a holistic summary of your health and fitness activities in one convenient place and can, more importantly, pair it up with the ongoing research.

The data obtained by the wearables and health app have to be well understood by the researcher because there are constraints on the quality of data collected. Take the sleeping data as an example. When using a wearable, the sleeping data are calculated not only by the non-usage of the wearable but also through heart rate measurement. Yet, when only using the phone as the data source, the phone takes into account a set of alarms and the non-usage of the phone to calculate these data. It may be challenging to understand the meaning of the resulting data. It may also be a challenge to give end users the appropriate information such that they understand what is being collected, how this data are used, and what are the ethical considerations when using these data.

#### Continuation

Apart from the functionalities of such a system, key to usage in practice is also the installation of a support engineering helpdesk, a product owner that prioritizes new developments, bug fixing and maintenance, explanatory materials such as e-learning videos, and the various technical disciplines needed, including DevOps, database administration, frontend, app, and backend development, product management, and business development.

## Results

Since 2016, over 350 studies have been conducted. This resulted in more than 40,000 questions answered. Studies have been developed by 320 researchers using TIIM including PhD candidates, students, researchers, and clinicians. All these were conducted in which data collection was powered by the platform and a vast body of thesis work. It is beyond the scope of this article to cover all in depth; however, we will describe four noteworthy use cases in digital literacy, mental health, resilience, and health coaching. The selection is based on peer-reviewed published cases that illustrate the evaluation and training possibilities of TIIM in daily life and (mental) health.

### Digital literacy investigation with proximity analysis in networks

In the studies by van der Zeeuw et al. (11, 12), the authors study the digital divide and (lack of) digital literacy in the Internet-of-Things (IoT) context. TIIM was used in combination with interview data as a diary study instrument to study socio-contextual dispositions as to why some people can better capitalize on IoT benefits creatively than others among 30 households. Among others, a special question type was introduced in TIIM, to support capturing of figurations (13). Figurations are networks of people but emphasize changing patterns of social and physical relations rather than their topology; they are the “flexible latticework of tensions.” In this study, human-IoT figurations were captured using TIIM, and the social “warmth” of the connections was also captured. This dimension indicates the added emotional value that fosters playfulness and creative use over its core functions.

The authors found (i) limitations caused by vendor lock-in become apparent when people use their IoT as a task-oriented tool for specific problems rather than considering the interoperability of IoT systems; (ii) that vendor lock-in is stimulating rather than hindering, but only if people have sufficient financial resources to integrate their IoT systems; and finally, (ii) that vendor lock-in can be considered a boundary and also a play-oriented challenge.

### Self-control training intervention

In the studies by Kip et al. (14, 15), the researchers developed and evaluated self-control training (SCT) using TIIM. Here, participants use their non-dominant hand for daily tasks. SCT is an effective intervention to increase both self-control and the behavior driven by self-control, such as reactive aggression. In contrast to an email-based alternative, analyses showed that the self-control of participants who used the app significantly improved over time [*F*(3, 196.315) = 4.090, *p* = 0.008]. Hence, the study showed that an SCT app has the potential to bolster self-control. Noteworthy, moreover, was that TIIM allowed for rapid prototyping and concept development and evaluation, which allowed the multidisciplinary team to visualize and discuss ideas with the stakeholders involved.

### Persuasive resilience coaching

In the studies by Lentferink et al. (16, 17), office workers were coached to increase their resilience and general condition. In total, 25% of office workers in Europe reported suffering from stress. TIIM was used to study (*n* = 28) office workers to deal with stress in an effective way. Specifically, the combination of self-tracking and persuasive e-coaching (18) was adopted to positively influence employees’ capacity for resilience. A 6-week “BringBalance” program allowed participants to perform reflection via four phases: (1) identification, (2) strategy generation, (3) experimentation, and (4) evaluation. Data collection consisted of log data, EMA reflection questionnaires through the e-Coach, in-depth interviews, and pre- and post-test surveys. Participants were able to perform self-reflection under the guidance of the automated e-Coach. This often led to gaining new insights. To improve the reflection process, more guidance should be offered by the e-Coach to aid employees in identifying events that actually recur in daily life.

### Digital self-isolation coaching

In the study by Van ‘t Klooster (19), a digital self-isolation electronic coach (e-Coach) app was built and evaluated during the COVID-19 pandemic. Its aim was to support individuals who had to either quarantine or self-isolate after a positive coronavirus test. The e-Coach, named IsolationCoach, gradually and conditionally provides relevant information (as opposed to conventional paper pamphlets/emails). Through a holistic patient view (19) and Socratic method (20) styled form of self-reflection, it provided emotional support to its users.

It was evaluated on the general Dutch people who self-isolated or self-quarantined (21) with regard to understandability, usability, outcome effects, and user experience. The studies elicited that participants found value in a digital coach who helped them comply with quarantine or isolation instructions and provided information on the practical challenges of organizing this. Also, usage of the app was appreciated. Third, participants experienced a need for mental support during their period of isolation or quarantine, which could partially be filled by the e-Coach through the Socratic method of self-reflection.

### Synthesis

The four case studies demonstrate the flexibility, usage, and adaptability of the platform. Published studies report on key elements of the platform. Specifically, custom question types and mobile questionnaires are used in van der Zeeuw’s study (11). Kip et al. (22) demonstrated the usage of TIIM for rapid prototyping in the mental health domain. The inclusion of vital sign monitoring and persuasive coaching is done in the study by Lentferink (17). Finally, van ‘t Klooster et al. (23) reported on an interactive coach using interactivity, calculated variables, and scheduling.

## Discussion

The usage of mobile coaching for prediction and prevention of adverse health outcomes is increasing, as is the importance of integrated supported self-management approaches. Strategic, technical, and operational investments are needed to allow versatile and rapid prototyping, development, implementation, and evaluation of app-based e-health solutions. We show that providing such an ecosystem allows for research and interventions in various healthcare contexts. The presented use cases in this article demonstrate that flexibility, adaptability and control-over-platform are important criteria when conducting e-health research using innovative interventions. As such, TIIM contributes to answering wicked e-health challenges.

### Challenges and opportunities

There is a plethora of research opportunities in mobile coaching (24). We discuss ones that specifically relate to including intervention mechanisms, fine-grained monitoring and inclusion of objective biomarker data.

#### Appropriately formulated and timed coaching

To provide successful coaching, just-in-time delivery of intervention components tailored to patients is key (25, 26). Although behavioral change takes time, the challenge of building up an effective relationship between the patient and the intervention requires an understanding of the context, a tailored intervention, the possibility to intervene at appropriate moments, and integrated strategies to optimize adherence (27, 28). Inevitably, this poses challenging requirements not only on the technology used and the delivery system but also on the system of experts involved and the sensing of the patient context. On the other hand, when successful in tackling this challenge, great healthcare benefits in large patient groups at low costs are possible. The patients themselves should however possess minimum digital literacy, smartphone experience, and mobile internet in order to use the service.

#### Inclusion of objective biomarker data

Currently, e-coaching systems generally do not measure or include objective biomarker data but rely on protocols and subjective information provided by the patient. Although fourth-generation EMI systems, as presented in this article, exist nowadays, there are still many opportunities to include biomarker data in measurement for research, patient profiling, and giving direct and validated feedback based on a combination of these objective data, especially when combined with the subjective information patients provide. Challenges here include system design, the privacy sensitivity of the data, collection and storage, interpretation of data measured in real-life settings, and the algorithm design as a basis for the feedback.

#### Advanced decision support

The advances of artificial intelligence (AI), especially large language models and generative AI, allow for novel applications and interaction methods. The e-coaching systems will benefit from these developments, as they allow for natural interaction, improved decision support and even decision making, and potentially better UX, for example, through natural interaction. Again, challenges here include the system and algorithmic design, the validation, andtaking an inclusive and transparent approach.

### Practical implications

The ecosystem presented already can be used in educational settings, treatment settings, and cohort and individual studies in (clinical) healthcare settings, and four universities and three hospitals have done so far.

The system can be used in new settings if the research or healthcare provider team has a vision of how the TIIM ecosystem could be beneficial. Both context experts and people familiar with e-health solutions should be available. The target end users should have a smartphone, internet, and basic digital literacy to install and subscribe to a new app. Finally, follow-up of usage by the main research team is important for adherence management.

The software is available upon reasonable request.

## Data Availability

The original contributions presented in the study are included in the article material, further inquiries can be directed to the corresponding author.
